# Six-Year Experience of a Nurse-Led Colorectal Cancer Follow-Up Clinic

**DOI:** 10.1155/2014/368060

**Published:** 2014-05-20

**Authors:** Hasan Al Chalabi, James M. O'Riordan, Alex Richardson, Delia Flannery, Katrina O'Connor, Charlotte Stuart, John Larkin, Paul McCormick, Brian Mehigan

**Affiliations:** Department of Colorectal Surgery, GEMS Directorate, Saint James' Hospital, Dublin 8, Ireland

## Abstract

*Aims and Objectives*. To review the experience of a nurse-led colorectal cancer follow-up clinic in a tertiary referral colorectal cancer centre. *Methodology*. Data from the nurse-led colorectal cancer follow-up clinic in our unit was prospectively maintained in a colorectal cancer database. Data was analysed from January 1, 2006 until the December 31, 2011. *Results*. 1125 patients were diagnosed with colorectal cancer, and referred to our unit as a tertiary centre for specialised colorectal cancer. Nine hundred and four patients had surgical resection of their colorectal cancer. Four hundred and seven patients were referred to the nurse-led colorectal cancer clinic for surveillance. The mean age of the patient cohort was 67 years (range 32–88) and 56% of patients were male. One hundred and seventeen patients were discharged to their general practitioner having been disease free after 5 years of followup. Fifty-four patients were diagnosed with either local or distant recurrence. *Conclusion*. A nurse-led colorectal cancer follow-up clinic is running according to strict follow-up protocols. This type of clinic significantly reduces the number of routine follow-up patients that have to be seen by the colorectal surgical consultant.

## 1. Introduction

The treatment of cancer in Ireland has changed over the last 5 years, with the introduction of centres of excellence in the Irish health service under the guidance of the National Cancer Control Programme [1]. The end result is 8 tertiary referral centres which now deal with all rectal cancers in Ireland. Taken with advances in neoadjuvant and adjuvant treatments and minimally invasive surgical techniques, survival of patients has improved following surgery for colorectal cancer [2–5]. This has led to an increasing number of patients being treated in these centres and subsequently requiring surveillance for several years following their treatment.

Followup for colorectal cancer patients following surgical resection of the primary tumour is the standard of care. It has been shown to be beneficial in terms of identifying potential patients with recurrence, either local or systemic, who might be candidates for either systemic chemotherapy and/or surgical resection of their recurrent disease [6–9]. The traditional model is that these patients would attend the consultant-led clinic and have their follow-up scans, tumour markers, and endoscopy arranged and the results checked at a subsequent visit. The increased concentration of the treatment of colorectal cancer in a smaller number of centres in Ireland has led to an increasing number of patients requiring surveillance in these centres. This has led to the introduction of nurse-led colorectal cancer follow-up clinics to help reduce the pressure on the consultant-led general surgical and colorectal outpatient clinics. In particular, consultants in these clinics are therefore able to devote more time and effort to the increasing number of new colorectal cancer patients. Also in the current financial environment with limited resources and economic pressure, the inception of nurse-led clinics can be seen as a saving model in the health sector where time and service could be focused and dedicated on other areas.

The aim of our study was to review our experience of a protocol driven nurse-led follow-up clinic for patients following surgery in a tertiary referral centre for colorectal cancer.

## 2. Methods

Data from the nurse-led colorectal cancer clinic in our unit is prospectively maintained by a full-time colorectal cancer data manager. Patients were analysed from January 1, 2006 until December 31, 2011. Data for all patients who underwent colorectal cancer surgery with curative intent during this time period was extracted.

**Figure 1:**
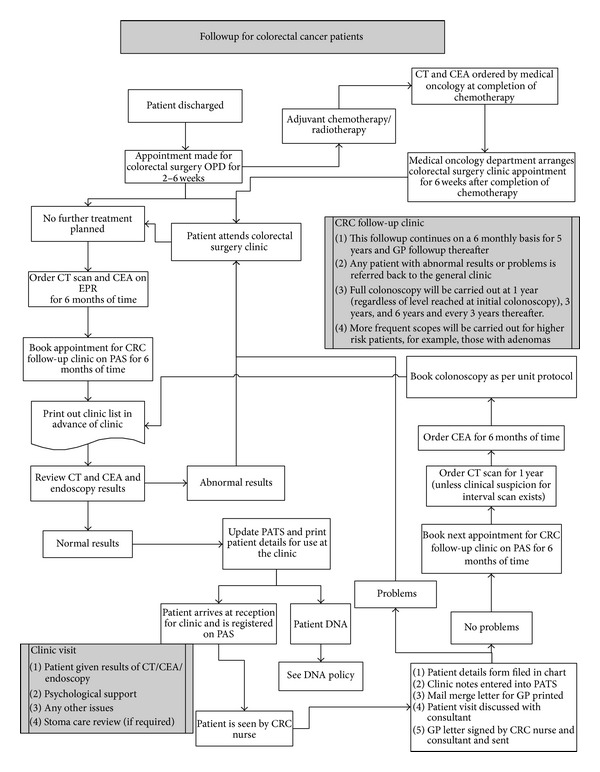
Demonstrates the follow-up protocol in the nurse-led colorectal cancer clinic.

The colorectal cancer nurse specialist who runs this clinic follows a strict protocol in terms of patient followup. This follow-up care pathway was designed by general consensus between the lead colorectal surgeons and the clinical nurse specialist in line with the international guidelines [10, 11]. All patients who underwent colorectal cancer surgery were followed up in the general surgical clinic; however, some were referred to nurse-led clinic; the followup is for at least 5 years.  Figure 1 demonstrates the protocol for followup that is adhered to in our unit. This pathway allows for consultant input whenever required and is designed to achieve a high standard of care. After the initial surgery, all patients are seen for their first postoperative visit by the consultant at 6 weeks following their surgery with the nurse-led specialist present for the consultation, and the patient is then referred to the nurse-led clinic for further surveillance if no adjuvant therapy is required. If adjuvant therapy was deemed necessary following the multidisciplinary meeting, those patients are then referred back to the general clinic after adjuvant treatment is completed and then to the nurse-led clinic as appropriate. The nurse-led protocol entails six monthly followups for 5 years, with 6-month CT scanning then yearly thereafter, and six monthly tumour markers (carcinoembryonic antigen), with colonoscopy at 1, 3, and 6 years. Medical and radiation oncology review are scheduled by the consultants if required following the outcome of the multidisciplinary team meeting and referrals are arranged if appropriate. If any concerning issues arise during surveillance, the patient is referred back to the consultant-led clinic for further assessment and management. Data from each visit is uploaded to the colorectal cancer database by the data manager to ensure accuracy and to allow future review and audit. Followup continues for up to 5 years postoperatively.

If patients are disease free after 5 years, they are discharged back to their general practitioner. Some patients, who are elderly and/or have comorbidities, are not or choose not to have followup and these patients are referred back to their family doctor. Other patients choose to have their followup in a private clinic or their locoregional hospital.

## 3. Results

**Figure 2:**
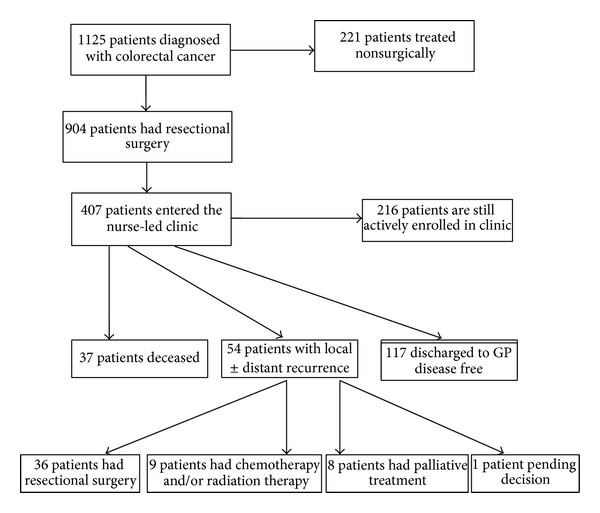
This demonstrates the flow of patients involved in the study.

From January 1, 2006 to December 31, 2011, one thousand one hundred and twenty-five patients were diagnosed with colorectal cancer in our unit or in other hospitals where patients were referred to our unit for further treatment. Nine hundred and four patients had surgical resection within this time period. The remaining two hundred and twenty one of these patients received nonsurgical treatment in our colorectal unit after a multidisciplinary meeting. Four hundred and seven patients who had colon or rectal cancer surgery were referred to the nurse-led colorectal cancer follow-up clinic (Figure 2). The rest were followed up in their local hospital, private hospitals, or local GPs. The mean age of these patients was 67 years, with a range of 32 to 88 years. Two hundred and twenty-eight patients were male (56%) and 179 (43%) were female. One hundred and seventeen patients were discharged back to their GP having been disease free for 5 years following their surgery.

Two hundred and sixteen patients are still actively engaged in the follow-up clinic at the time of our analysis.  Table 1 demonstrates the management of patients with disease recurrence. Thirty-seven patients died during the study period, of whom 20 patients died while attending the nurse-led clinic. Fourteen patients died from noncancer related causes. Fifty-four patients were diagnosed with either local or distant recurrence. Eighteen patients out of these 54 were still alive at the time of the analysis. Eight patients underwent lung resection; twelve patients underwent liver resection, while sixteen patients had a further colorectal resection for either an anastomotic recurrence or a metachronous tumour. Nine patients received either chemotherapy or radiation therapy alone, while eight patients were referred to the palliative care service for the management of incurable disease. One patient was still awaiting final decision regarding his cancer recurrence at the time of the study analysis.

## 4. Discussion

Colorectal cancer is the second most common cancer in Ireland with over 2,250 cases diagnosed in 2009 [12]. It is well documented in the literature that clear follow-up protocols for patients postoperatively are important in terms of outcome and survival [1, 6]. The pattern of postoperative care and surveillance is fundamental to detect early cancer recurrence as an ever increasing number of patients are referred for resectional surgery with liver and/or lung metastases. In addition, ongoing surveillance adds a feeling of confidence and security for patients in the postoperative setting [13].

A number of randomised controlled trials have demonstrated that intense followup after surgery for colorectal cancer can improve survival by up to 7% as disease recurrence is detected earlier than if no follow-up protocol is in place [14–16]. Figueredo et al. in a systematic review showed that earlier pickup of recurrent disease with an intense follow-up programme ultimately leads to higher rates of reoperation with curative intent [14]. It is well established that hepatic resection of colorectal liver metastases in either the combined or staged setting is feasible with reduced morbidity and associated with improved survival [17]. Stiggelbout et al. also demonstrated that regular outpatient visits with structured followup have a favourable impact for patients in terms of reassurance and reducing anxiety [18]. Other studies also demonstrated that the benefits achieved by an intense follow-up regime are not purely related to earlier detection of cancer recurrence but may also be related to better psychological support, improved diet and lifestyle adjustments, and earlier treatment of any coincidental diseases [19].

Our study summarises our initial experience of a nurse-led colorectal cancer clinic since its inception six years ago. It has proven to be an effective and efficient means from medical and financial aspects of identifying patients with recurrent disease and referring suitable patients following mutidisciplinary team discussion for further resectional surgery. We identified a total of 54 patients with either local and/or distant recurrent disease, 36 of whom underwent a second surgical intervention with curative intent. There is no doubt that the increasing pressure on the consultant-led clinic in terms of general surgery and colorectal surgery work load has mandated the establishment of a nurse-led clinic, particularly in an era where rectal cancer surgery in Ireland is increasingly practiced in a smaller number of centres.

It is described by some authors that the time allocated to follow-up patients in nurse-led colorectal cancer clinics is far longer than the time allocated in a consultant-led surgical clinic [20–22]. We agree with this finding and it is also our experience that a close and personable relationship is built up between patients and the colorectal cancer nurse during the follow-up period with resultant high patient satisfaction rates. The role of the nurse is to help patients in terms of education about their disease, provide guidance and support for patients and family members, and facilitate interaction with other disciplines such as stoma care and dietetics and our social work department.

In conclusion, our experience of a nurse-led follow-up clinic is that it is an efficient and effective means of followup for patients following surgery for colorectal cancer. It significantly reduces the number of patients that the consultant has to see while at the same time, through the use of a standardised protocol, provides an excellent method of detecting patients with recurrent disease and referring those patients for further management.

## Figures and Tables

**Table 1:** Breakdown of the management of 54 patients who were diagnosed with recurrent disease in the nurse led follow-up clinic.

Site of recurrence	Treatment received	No of patients
Colon	Right Colon resection	3
	Left Colon resection	10
	Colon resection + Chemotherapy	3
Total		**16**
Lung	Lung resection	3
	Lung resection + Chemotherapy	5
Total		**8**
Liver	Right segmental liver resection	2
	Left segmental liver resection	2
	Liver resection + chemotherapy	8
Total		**12**
Distant metastasis	Chemotherapy + radiotherapy	5
	Curative intent radiotherapy alone	4
	Palliative treatment	8
Total		**17**
Pending decision		**1**

Total no of patients received treatment		54
